# Effects of Phospholipase A_2_ and Metalloproteinase Inhibitors on Skeletal Muscle Regeneration After Myonecrosis Induced by the Venom of *Bothrops asper*: An Initial Assessment in Mice

**DOI:** 10.3390/toxins18090359

**Published:** 2026-08-23

**Authors:** Andrés Zamora, Alexandra Rucavado, Teresa Escalante, José María Gutiérrez, Erika Camacho

**Affiliations:** 1 Instituto Clodomiro Picado, Facultad de Microbiología, Universidad de Costa Rica, San José 11501, Costa Rica; andres.zamoraramirez@ucr.ac.cr (A.Z.); alexandra.rucavado@ucr.ac.cr (A.R.); teresa.escalante@ucr.ac.cr (T.E.); 2 Departamento de Fisiología, Escuela de Medicina, Universidad de Costa Rica, San José 11501, Costa Rica

**Keywords:** *Bothrops asper* venom, myonecrosis, muscle regeneration, fibrosis, Varespladib, Marimastat

## Abstract

Skeletal muscle regeneration is often impaired after acute muscle damage induced by viperid snake venoms, such as that of *Bothrops asper*, a medically relevant species in Latin America. It has been shown that traces of venom that remain in the damaged muscle affect myogenic cells in culture, raising the possibility of inhibiting these toxins during the regenerative process to improve regeneration. Using a mouse model of myonecrosis and regeneration, we evaluated the effects of Varespladib (a phospholipase A_2_ inhibitor) or Marimastat (a metalloproteinase inhibitor) on muscle regeneration when administered intravenously 24 h after the onset of myonecrosis, i.e., after muscle damage has occurred. The regenerative process was evaluated 14 and 28 days after venom injection. Marimastat, or a combination of both inhibitors, increased the number and diameter of regenerating muscle fibers and reduced tissue fibrosis compared to tissues from mice receiving only venom, as judged by qualitative and quantitative histological assessment. These initial results underscore the deleterious role of traces of venom components, especially metalloproteinases, in damaged muscles during muscle regeneration.

## 1. Introduction

Envenomings by viperid snakes are characterized by local and systemic pathological and pathophysiological manifestations, including necrosis, hemorrhage, edema, blistering, coagulopathy, systemic bleeding, acute kidney injury and cardiovascular shock [1,2,3]. These envenomings often result in pronounced damage to skeletal muscle, i.e., myonecrosis, which may result in permanent tissue damage [1,2,3]. *Bothrops asper* is medically the most important snake in Central America. Envenomings by this species are characterized, among other clinical features, by muscle tissue damage, i.e., myonecrosis. This effect is the result of the action of myotoxic phospholipases A_2_ (PLA_2_s) and PLA_2_ homologs, which directly damage muscle fibers, and hemorrhagic metalloproteinases (SVMPs), which induce ischemia [1,4]. Skeletal muscle has an intrinsic capacity to regenerate after several types of injury [5]. However, in the case of viperid venoms, such as that of *Bothrops asper*, and hemorrhagic SVMPs, the regenerative response is often impaired [6,7,8,9], with the consequent loss of muscle mass and its replacement by fibrotic and adipose tissues. This results in long-term sequelae which drastically affect the quality of life of snakebite victims [1,10]. In contrast, regeneration is successful after myonecrosis induced by toxins and venoms that affect muscle fibers without damaging the vasculature, such as those of snakes of the family Elapidae [6,8,11,12]. The reasons behind such a poor regenerative outcome in the case of viperid venoms are not completely understood, but are likely to be multifactorial, involving damage to the microvasculature and intramuscular nerves, deficient removal of necrotic debris, and degradation of the basement membrane of muscle fibers, among other mechanisms [6,8,13].

When investigating the effect of homogenates of skeletal muscle obtained from mice after myonecrosis induced by *B. asper* venom or isolated muscle-damaging toxins, it was found that these homogenates inhibited myoblast replication and/or fusion into myotubes in cell culture [14]. Similar observations were described with the venoms of other *Bothrops* species [15]. Since such effects were abrogated when the homogenates were incubated with antivenom, it was concluded that traces of venom in the regenerating tissue were responsible for affecting muscle myogenic cells. Moreover, the venoms of the viperids *Daboia russelii* and *Bitis arietans* are also known to affect myoblast viability, to inhibit their fusion into myotubes, and to induce atrophy of myotubes in culture [16,17]. Thus, traces of venom remaining in muscle tissue after myonecrosis may affect regenerating muscle cells and contribute to the impairment of the regenerative process in envenomings by a variety of viperid species. This raises the possibility of using inhibitors of muscle-damaging toxins along the regenerative process after acute myonecrosis as a therapeutic tool to enhance regeneration.

Since myotoxic phospholipases A_2_ (PLA_2_s) and hemorrhagic metalloproteinases (SVMPs) play key roles in acute muscle damage induced by *B. asper* venom [4,18], and homogenates from muscle tissue injected with these toxins impair myoblast fusion to form myotubes [14], we hypothesized that the administration of inhibitors of these enzymes, i.e., Varespladib and Marimastat, after the development of local myonecrosis, would result in improved skeletal muscle regeneration. An initial assessment of this hypothesis is presented in this work.

## 2. Results

### 2.1. Muscle Necrosis Induced by B. asper Venom

**Figure 1:**
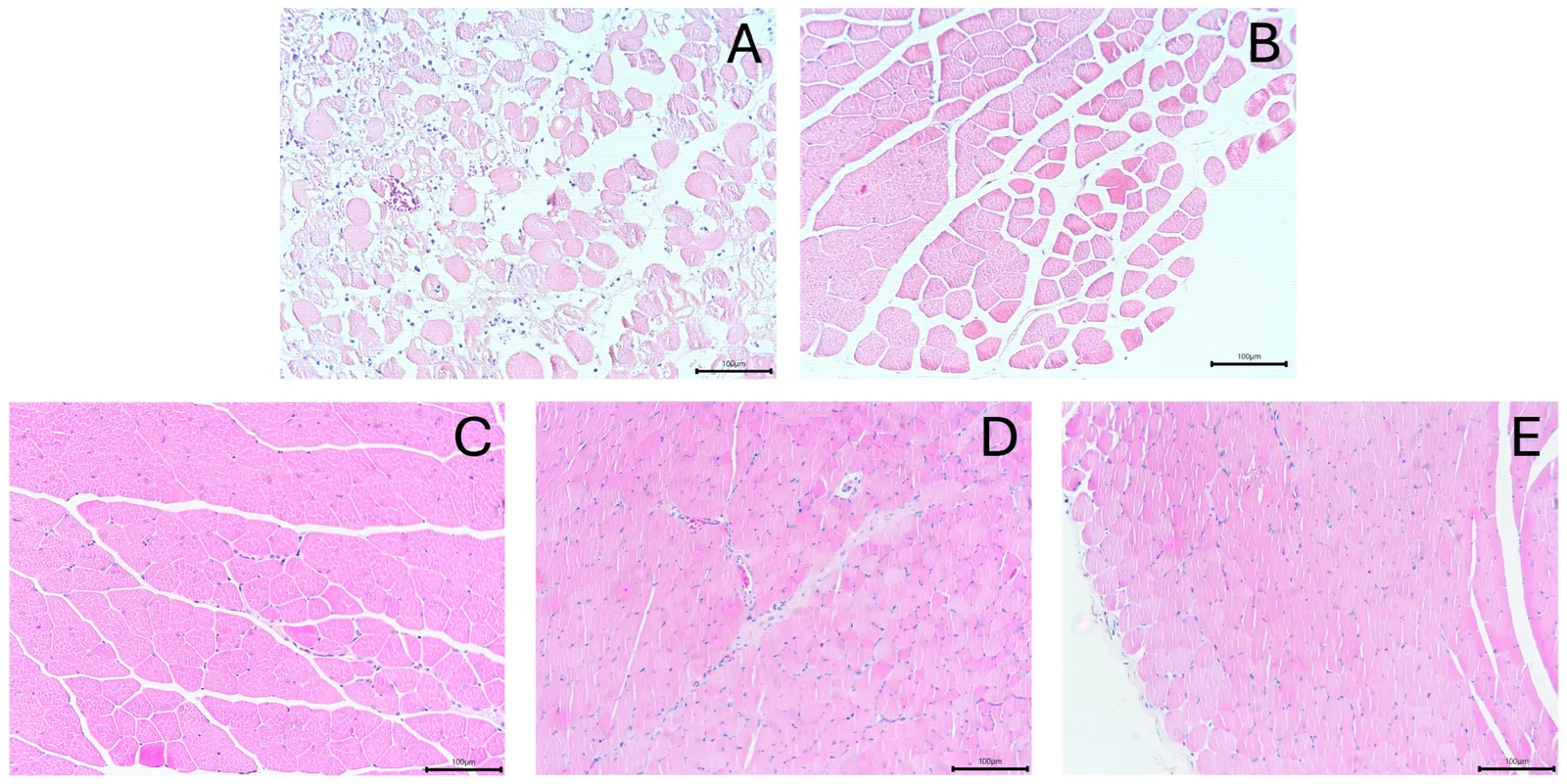
Light micrographs of sections of mouse gastrocnemius muscles 24 h after intramuscular injection of 50 µg *B. asper* venom (**A**), PBS (**B**), or intravenous injection of 80 µg Varespladib (**C**), 80 µg Marimastat (**D**), or 80 µg Varespladib + 80 µg Marimastat (**E**). Normal histological appearance of skeletal muscle is observed in (**B**–**E**), whereas widespread necrosis of muscle fibers and inflammatory infiltrates are observed in (**A**). Hematoxylin–eosin staining. Bars represent 100 µm.

To assess the extent of skeletal muscle damage induced by the intramuscular (i.m.) injection of 50 µg *B. asper* venom in the gastrocnemius muscle, tissue sections were analyzed 24 h after venom injection, since previous studies demonstrated that tissue damage induced by *B. asper* venom occurred within the first hours after injection [18,19,20]. Thus, assessing tissue necrosis at 24 h allowed the quantification of the net extent of muscle damage. Widespread necrosis of muscle fibers, extravasated erythrocytes, and abundant inflammatory cells were observed (Figure 1A), which are characteristic pathological alterations induced by this venom [20]. The muscle necrotic area, characterized by hypercontracted muscle fibers with fragmented cytoplasmic material, corresponded to 48.2 ± 13.7% of the total area of muscle (Table S1A). This observation agrees with the value of 50% necrosis found in a previous study using the same experimental model with this venom [19]. With this basis, we then assessed the effect of inhibitors on skeletal muscle regeneration when administered 24 h after venom. In contrast, tissue samples from control mice injected i.m. with PBS, or i.m. with PBS followed, 24 h later, by intravenous (i.v.) administration of either PBS, Varespladib, Marimastat, or Varespladib + Marimastat showed normal histological appearance of muscle tissue, with no alterations observed in muscle fibers, blood vessels or nerves, and with no necrotic fibers (Figure 1B–E). Moreover, no fibrosis was observed in these samples from control mice receiving PBS only or PBS followed by the inhibitors. Thus, the inhibitors did not induce histological alterations in muscle tissue.

### 2.2. Effect of Varespladib and Marimastat on Skeletal Muscle Regeneration After Necrosis Induced by B. asper Venom

#### 2.2.1. Observations in Samples Collected 14 Days Post-Envenoming

**Figure 2:**
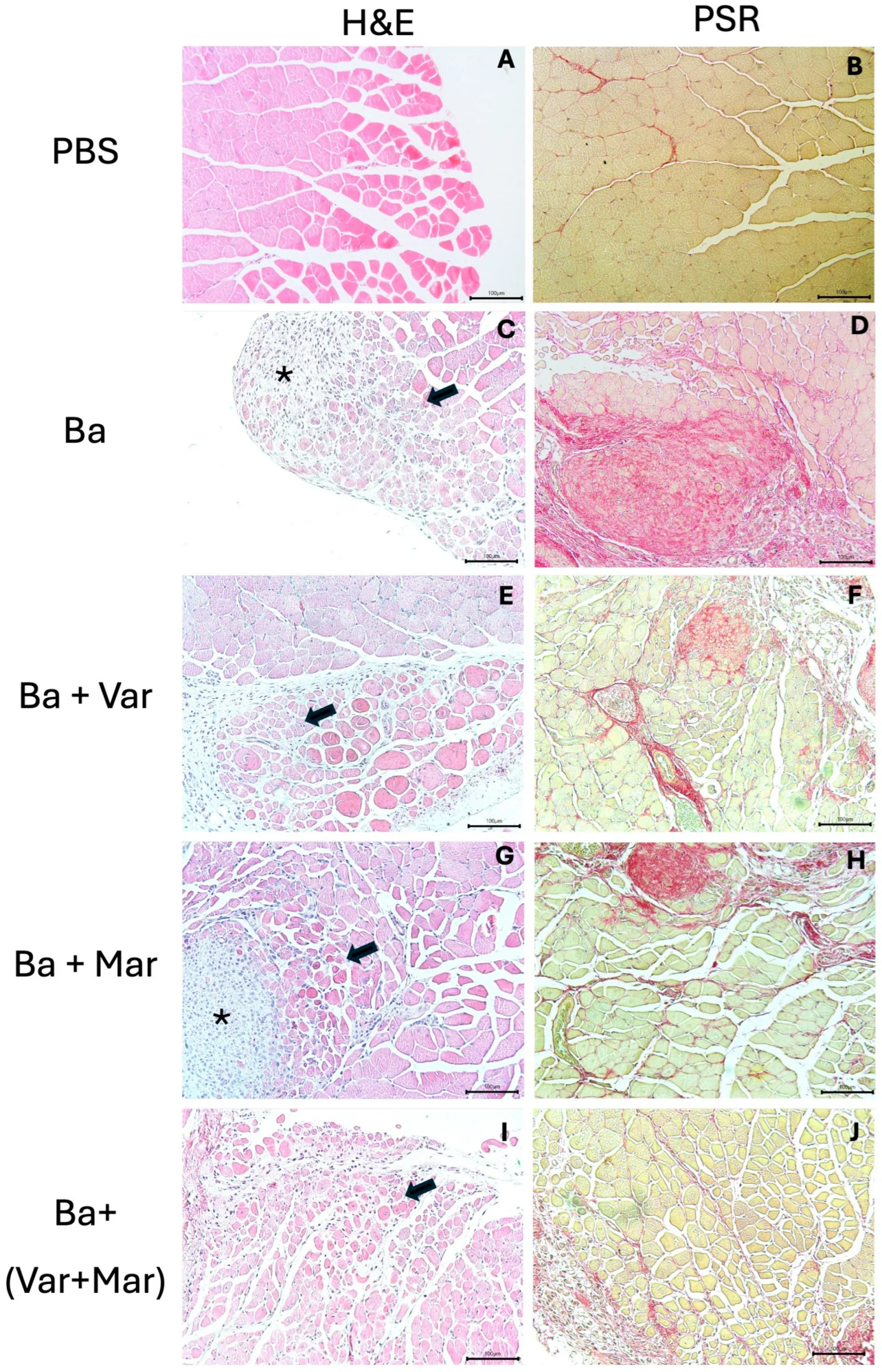
Light micrographs of sections of mouse gastrocnemius muscles 14 days after injection of venom followed by either PBS or inhibitors. Mice received an i.m. injection of 50 µg *B. asper* venom or PBS. Then, 24 h after envenoming, different groups received an i.v. injection of either PBS (Ba, (**C**,**D**)), Varespladib (Ba + Var, (**E**,**F**)), Marimastat (Ba + Mar, (**G**,**H**)), or Varespladib + Marimastat (Ba + Var + Mar, (**I**,**J**)). The control group was injected i.m. with PBS and then received PBS i.v. after 24 h (PBS, (**A**,**B**)). Deficient muscle regeneration was observed in sections from envenomed mice not treated with inhibitors. Sections from tissues of envenomed mice treated with the inhibitors showed evidence of improved muscle regeneration and a lesser extent of fibrosis. Arrows represent areas of regenerating muscle fibers with centrally located nuclei and asterisks represent areas of poor regeneration. H&E: hematoxylin–eosin staining; PSR: picrosirius red staining. Bars represent 100 µm.

Tissue sections collected 14 days after the injection of PBS showed a normal histological pattern of muscle tissue, without fibrosis, and were devoid of muscle fibers with centrally located nuclei, i.e., regenerating fibers (Figure 2A,B). On the other hand, at this time interval, the tissue of mice that received *B. asper* venom and then PBS instead of inhibitors showed scarce and dispersed areas of muscle regeneration, characterized by small muscle fibers with centrally located nuclei, and areas where fibrosis and granulation tissue predominated (Figure 2C). In contrast, sections from the muscle of mice that were treated with either Varespladib or Marimastat 24 h after venom injection showed a histological pattern characterized by a higher number of regenerating muscle fibers with centrally located nuclei and fewer areas of poor regeneration (Figure 2E,G). This trend was more pronounced in samples from envenomed mice treated with the combination of Varespladib and Marimastat (Figure 2I). Tissue sections stained with picrosirius red evidenced a high extent of fibrosis in samples from envenomed mice not receiving the inhibitors (Figure 2D), whereas less abundant picrosirius-red-stained areas were observed in samples of envenomed mice receiving the inhibitors (Figure 2F,H,J).

**Figure 3:**
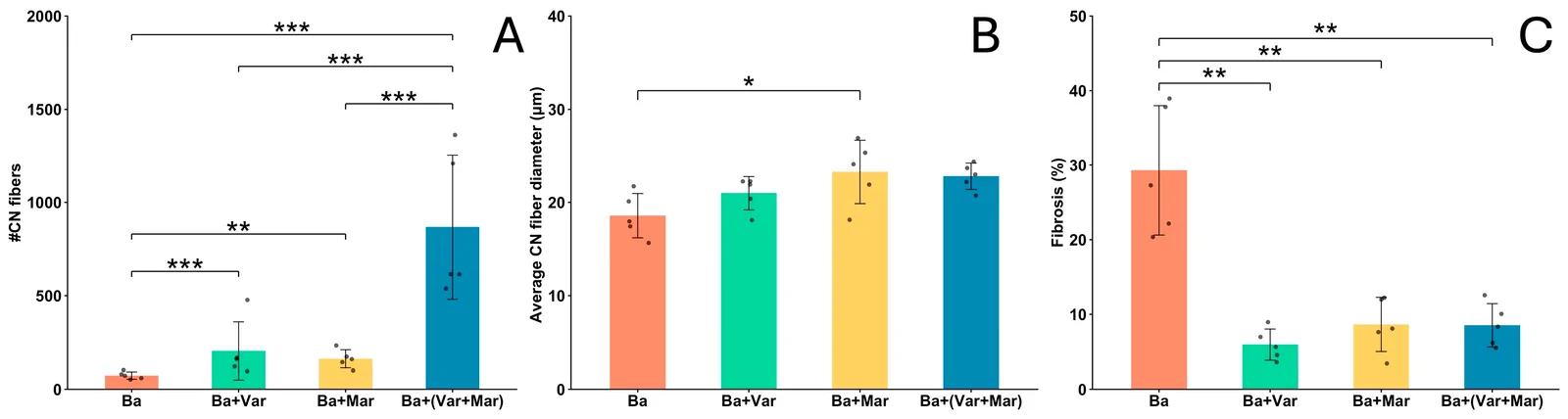
Quantitative histological assessment of skeletal muscle regeneration 14 days after injection of *B. asper* venom, followed 24 h later by the i.v. injection of either PBS (Ba), Varespladib (Ba + Var), Marimastat (Ba + Mar), or Varespladib + Marimastat (Ba + Var + Mar). Tissue sections were stained with hematoxylin–eosin or picrosirius red. (**A**) Number of fibers with centrally located nuclei (CN) in the whole muscle section. (**B**) Average diameter of the totality of muscle fibers having centrally located nuclei (CN). (**C**) Percentage of the total area of muscle showing predominance of picrosirius red staining. Statistically significant differences were determined by negative binomial regression (**A**), one-way ANOVA with Tukey *post hoc* test (**B**), and a general linear model (GLM) with gamma distribution (**C**). Values represent mean ± S.D. (*n* = 5). * *p* < 0.05; ** *p* < 0.01; *** *p* < 0.001.

To provide a quantitative assessment of these observations, we evaluated the extent of regeneration by assessing the number of regenerating muscle fibers per muscle and the diameter of the regenerating fibers. All treatments with inhibitors induced a significant increase in the number of regenerating fibers, i.e., fibers with centrally located nuclei, per muscle 14 days after envenoming. With both inhibitors combined, the increase in the number of regenerating fibers was higher than when each inhibitor was administered alone (Figure 3A). On the other hand, no significant differences were observed in the diameter of regenerating fibers between treatments, except for the envenomed group treated with Marimastat, which showed a significant increase in the diameter of regenerating fibers compared to samples from envenomed mice that did not receive inhibitors (Figure 3B). Regarding fibrosis, assessed by staining with picrosirius red, tissue from envenomed mice receiving Varespladib, Marimastat, or Marimastat + Varespladib showed a significant reduction in the extent of fibrotic areas of the muscle compared to samples from mice receiving venom and no inhibitors (Figure 3C). The raw data used to prepare the graphs of Figure 3 are included in Table S1B.

#### 2.2.2. Observations in Samples Collected 28 Days Post-Envenoming

**Figure 4:**
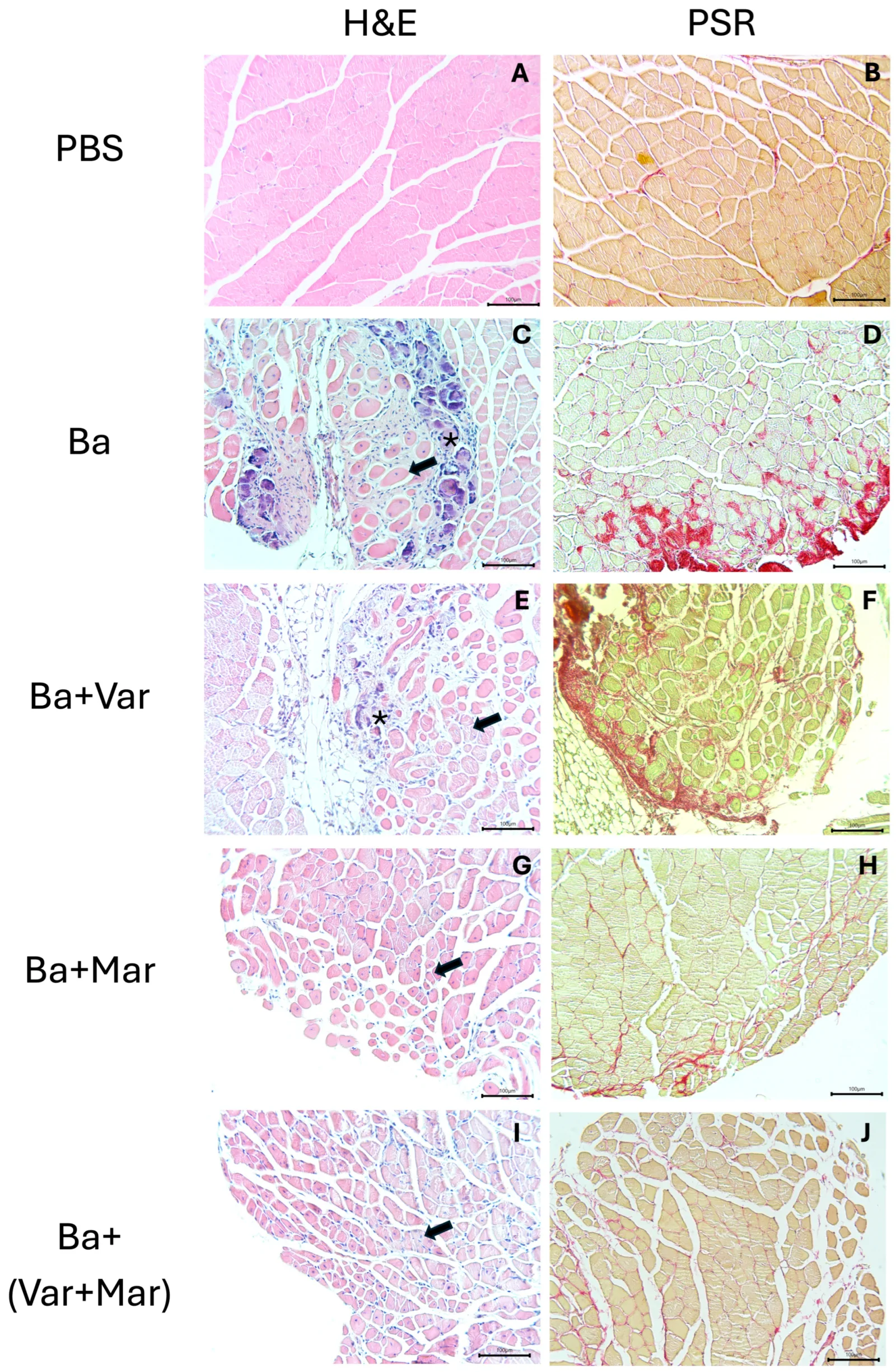
Light micrographs of sections of mouse gastrocnemius muscles 28 days after injection of PBS or venom followed by either PBS or inhibitors. Mice received an i.m. injection of 50 µg *B. asper* venom or PBS. Then, 24 h after envenoming, different groups received an i.v. injection of either PBS (Ba, (**C**,**D**)), Varespladib (Ba + Var, (**E**,**F**)), Marimastat (Ba + Mar, (**G**,**H**)) or Varespladib + Marimastat (Ba + Var + Mar, (**I**,**J**)). The control group was injected i.m. with PBS and then received PBS i.v. after 24 h (PBS, (**A**,**B**)). Deficient muscle regeneration is observed in sections from envenomed mice not treated with inhibitors. Sections from tissues of envenomed mice treated with the inhibitors, especially with Marimastat or Marimastat + Varespladib, showed evidence of improved muscle regeneration and a lesser extent of fibrosis. Arrows represent areas of regenerating muscle fibers with centrally located nuclei, whereas asterisks represent areas of poor regeneration. H&E: hematoxylin–eosin staining; PSR: picrosirius red staining. Bars represent 100 µm.

Tissue sections collected 28 days after the injection of PBS showed a normal histological pattern of muscle tissue, without fibrosis, and were devoid of muscle fibers with centrally located nuclei, i.e., regenerating fibers (Figure 4A,B). In order to assess whether the administration of the inhibitors in non-envenomed mice has an impact on the morphometric parameters used in this study, tissue samples from mice receiving an i.m. injection of PBS followed 24 h later by an i.v. injection of either Varespladib, Marimastat or Varespladib + Marimastat were analyzed. No regenerating fibers, i.e., fibers with centrally located nuclei, were observed in these samples, in agreement with the lack of myotoxicity induced in these controls. The diameter of muscle fibers was assessed in 400 fibers per treatment. Diameters corresponded to 42.6 ± 6.6 µm, 41.8 ± 7.3 µm, and 40.6 ± 7.4 µm in samples from mice receiving Varespladib, Marimastat or Varespladib + Marimastat, respectively. These values did not differ significantly (*p* > 0.05) from the diameters of muscle fibers in controls receiving only PBS (40.7 ± 8.3 µm). These findings corroborated the lack of deleterious effects of the inhibitors in skeletal muscle in the experimental conditions of this work. Moreover, no areas of fibrosis were observed in tissue sections from these samples stained with picrosirius red.

When the analyses were carried out in tissue samples from envenomed mice collected 28 days after envenoming, a significant increase in the wet weight of the injected gastrocnemius muscle was observed in envenomed mice that received Marimastat compared to those that did not receive inhibitors, whereas no significant differences were observed with the other treatments. The weights of muscles from all envenomed groups were significantly different from that corresponding to mice receiving PBS and no venom, except for the envenomed group treated with Marimastat (Figure S1 and Table S1C). Some mice died in these groups in the time lapse between 14 and 28 days after venom injection. The numbers of dead mice were one, two, 0, and one in the venom control, venom + Varespladib, venom + Marimastat, and venom + Varespladib + Marimastat groups, respectively.

Sections from samples of mice injected with venom and receiving PBS instead of inhibitors showed a mixture of areas of regenerative muscle fibers of small diameter and areas of poor regeneration characterized by fibrotic tissue and regions showing apparent calcification (Figure 4C). In the cases of samples from envenomed mice treated with inhibitors, especially those receiving Marimastat or Marimastat + Varespladib, there was a better regenerative landscape, with centrally nucleated muscle fibers intermixed with fewer areas of fibrosis (Figure 4E,G,I). The assessment of fibrosis by picrosirius red staining evidenced an apparent reduction in interstitial collagen in all samples compared to those collected at 14 days. However, tissue from envenomed mice not treated with inhibitors or treated with Varespladib showed a higher extent of picrosirius staining (Figure 4D,F) when compared to samples from envenomed mice that received the inhibitors, especially Marimastat or Marimastat + Varespladib (Figure 4H,J).

**Figure 5:**
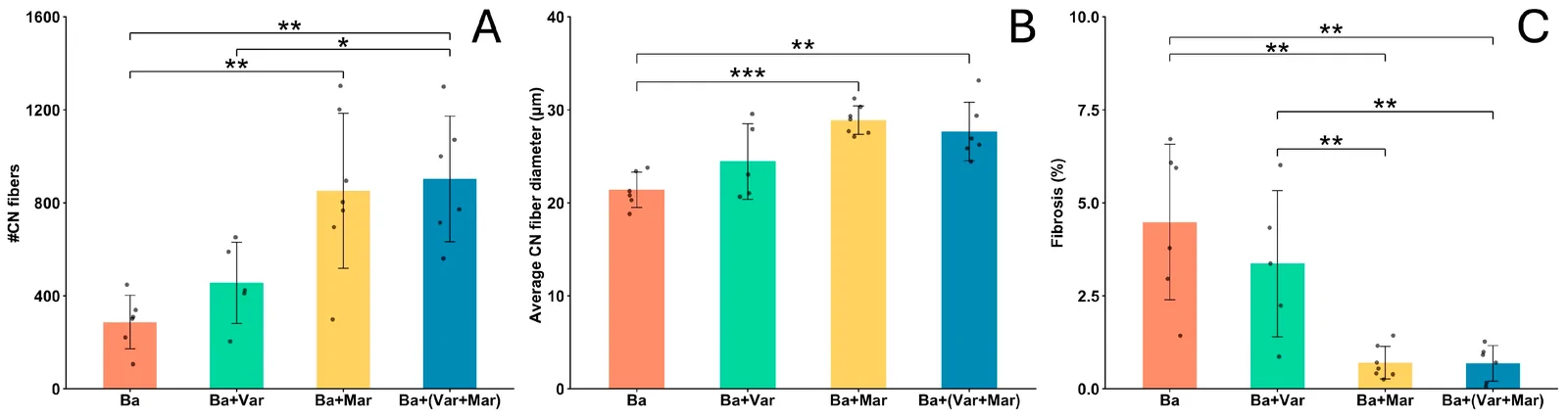
Quantitative histological assessment of skeletal muscle regeneration 28 days after injection of *B. asper* venom, followed 24 h later by the i.v. injection of either PBS (Ba), Varespladib (Ba + Var), Marimastat (Ba + Mar) or Varespladib + Marimastat (Ba + Var + Mar). Tissue sections were stained with hematoxylin–eosin or picrosirius red. (**A**) Number of fibers with centrally located nuclei (CN) in the whole muscle section. (**B**) Average diameter of the totality of muscle fibers having centrally located nuclei (CN). (**C**) Percentage of the total area of muscle showing predominance of picrosirius red staining. Statistically significant differences were determined by one-way ANOVA with Tukey *post hoc* test (**A**,**B**) and a general linear model (GLM) with gamma distribution (**C**). Values represent mean ± S.D. (*n* = 5–7). * *p* < 0.05; ** *p* < 0.01; *** *p* < 0.001.

When a quantitative assessment of muscle regeneration was carried out in samples collected at 28 days, tissue from envenomed mice that received Marimastat or Marimastat + Varespladib showed a significantly higher number of regenerating fibers compared to mice injected with venom and no inhibitors (Figure 5A). At this time interval, no significant improvement in regeneration was observed in the group of envenomed mice receiving Varespladib (Figure 5A). In agreement, a significantly higher diameter of regenerating fibers was observed in samples of envenomed mice treated with Marimastat or Marimastat + Varespladib, but not in those receiving Varespladib only, when compared to samples from envenomed mice that did not receive inhibitors (Figure 5B). The extent of intramuscular fibrosis, as judged by picrosirius red staining, was reduced in all samples when compared to those collected at 14 days. When compared to samples at 28 days from envenomed mice not receiving inhibitors, the percentage of fibrotic areas was reduced in samples from mice treated with Marimastat or Marimastat + Varespladib, but not in the case of envenomed mice receiving only Varespladib (Figure 5C). The raw data used to prepare the graphs of Figure 5 are included in Table S1D.

## 3. Discussion

Our findings show that i.v. administration of Marimastat, or a combination of Marimastat and Varespladib, after the development of muscle necrosis induced by *B. asper* venom results in an improvement of the muscle regenerative response. In this experimental setting, the beneficial effects of these inhibitors are not due to the inhibition of venom-induced acute tissue damage, which has already occurred by 24 h. Instead, the observed effect is likely caused by the inhibition of venom or endogenous enzymes present in the tissue during the early stages of muscle regeneration. Previous studies have shown that traces of venom components remaining in the affected tissue have deleterious effects on myogenic cells [14,15], thus supporting the hypothesis that the inhibition of SVMPs in the tissue improves the regenerative outcome. In our experimental model, the improvement in regeneration was only partial, since the diameter of regenerating fibers did not achieve the diameter of mature muscle fibers in control samples from mice receiving either PBS or the inhibitors alone.

Varespladib and Marimastat were selected as inhibitors because they inhibit snake venom PLA_2_s, in the case of Varespladib, and SVMPs, in the case of Marimastat, in a variety of snake venoms [21,22,23,24,25,26]. Varespladib reduced the extent of muscle damage and improved muscle regeneration after injection of the venom of *Deinagkistrodon acutus* [25]. Moreover, Varespladib and Prinomastat, another metalloproteinase inhibitor, abrogated atrophy induced by the venom of *D. russelii* on myotubes in culture [16].

Marimastat, and especially the combination of both inhibitors, showed the strongest pro-regenerative effect. It was of interest that the combination induced a marked increase in the number of regenerating fibers by 14 days, whereas by 28 days the treatments based on Marimastat or the combination of inhibitors resulted in a similar improvement. These findings suggest that SVMPs remaining in the tissue exert a stronger inhibitory role in the regenerative process than PLA_2_s. However, the fact that the combination of Marimastat and Varespladib induced a higher extent of regeneration at 14 days, as judged by the number of regenerating cells in the damaged muscle, suggests that they may be acting synergistically in promoting the early stages of regeneration. On the other hand, the observation that the number of regenerating fibers is similar at 14 days in samples of mice treated with the combination of inhibitors and at 28 days in samples of mice treated with Marimastat or with the combination suggests that muscle regeneration reaches a plateau and that it is accelerated when the two inhibitors are used together.

Varespladib was originally designed to inhibit endogenous PLA_2_s for the treatment of rheumatoid arthritis, sepsis and acute coronary syndrome [27,28,29] and Marimastat for inhibiting endogenous matrix metalloproteinases (MMPs) in vascular disease and malignancies [30,31]. Thus, the possible inhibition of endogenous enzymes in muscle tissue having a role in muscle repair and regeneration cannot be discarded. MMPs are known to play key roles in muscle repair and regeneration [32,33,34] and their activities increase in muscle injected with PLA_2_ and SVMP from *B. asper* venom [35]. MMPs also play a role in the regulation of muscle resident fibro/adipogenic precursors which proliferate and give rise to adipocytes in various muscle pathologies [36]. Therefore, the inhibitors used in our study may be working not only against snake venom enzymes but also against endogenous enzymes, which is an issue that requires further study.

The mechanisms by which *B. asper* venom PLA_2_s and SVMPs affect muscle regeneration remain unknown. Myotoxic PLA_2_s are cytotoxic to myoblasts and myotubes in culture [37,38], and apoptotic muscle-regenerating cells have been described in regenerating muscle after myonecrosis induced by *B. asper* venom [8]. On the other hand, SVMPs affect myogenic satellite cells [39] and degrade the basement membrane that surrounds muscle fibers, which plays a key role in the regenerative response [39,40]. Moreover, since revascularization is relevant during muscle regeneration, the damaging effect of hemorrhagic SVMPs on the microvasculature [8,41] may also affect angiogenesis. In our experimental setting, the inhibitors were administered by the i.v. route 24 h after venom injection, a time when the activation and proliferation of myogenic cells in damaged muscle are underway. Thus, the inhibitors are likely to block venom SVMPs and PLA_2_s by the time key steps in the regenerative process are taking place.

Our study has several limitations. Only one dose of the inhibitors was used, and they were administered at only one time point (24 h after venom injection). Furthermore, our assessment of regeneration was based on histological/morphological parameters, and no functional evaluation was carried out. The observed improvement in the regenerative response was only partial, since the diameter of regenerating fibers did not reach the diameter or normal muscle fibers, and there was residual fibrosis as well. However, our findings show that Marimastat, administered at the dose and time described, improves the regenerative process, as per the histological parameters analyzed.

In conclusion, our findings provide clues to understand one of the mechanisms responsible for the poor muscle regeneration after myonecrosis induced by viperid snake venoms. The results support the concept that venom toxins remaining in the tissue during the first stages of muscle regeneration may affect the regenerative process. The beneficial role of Marimastat, or the combination of Marimastat and Varespladib, suggests that SVMPs may be involved in such an effect by directly damaging myogenic precursors or hydrolyzing the extracellular matrix components necessary for regeneration. Future studies should test whether other protocols (repetitive administrations and variable doses of the inhibitors, or the use of alternative inhibitors or their combinations) result in further improvements in muscle regeneration.

## 4. Materials and Methods

### 4.1. Venom and Inhibitors

The venom of the snake *Bothrops asper* was used throughout this study. The venom was a pool obtained from more than 40 adult specimens collected in the Pacific region of Costa Rica and maintained at the serpentarium of Instituto Clodomiro Picado. After lyophilization, venom was stored at −20 °C. Venom solutions were prepared in 0.12 M NaCl and 0.04 M phosphates at pH 7.2 (PBS) immediately before use. The inhibitor Varespladib (LY315920) was provided by Ophirex, Inc. (Corte Madera, CA, USA) via ChemiTek (Indianapolis, IN, USA), and Marimastat (BB-2516) was from Sigma_Aldrich (Sigma-Aldrich, St. Louis, MO, USA).

### 4.2. Experimental Animals

Throughout this study, mice (CD-1 strain, females, 18–20 g body weight) were used. Mice were provided by the bioterium of Instituto Clodomiro Picado. They were housed in plastic cages, kept under controlled temperature and a 12:12 h light–dark cycle, and provided with water and food *ad libitum*. Throughout the study, mice were allocated randomly in cages by the staff of the bioterium. The protocol of experiments involving animals was approved by the Institutional Committee for the Care and Use of Animals (CICUA) of the University of Costa Rica (approval code CICUA-17-2023). All procedures followed the International Guiding Principles for Biomedical Research involving Animals (CIOMS).

### 4.3. Quantification of Muscle Tissue Necrosis Induced by B. asper Venom

For the assessment of the extent of muscle tissue necrosis induced by *B. asper* venom at 24 h, a group of eight mice received an intramuscular (i.m.) injection, in the right gastrocnemius, of 50 µg venom dissolved in 50 µL PBS. A control group of mice received 50 µL PBS, under otherwise identical conditions. Additional control groups included mice that received 50 µL PBS i.m. followed, 24 h later, by the intravenous (i.v.) injection of 100 µL of either Varespladib (0.8 mg/mL), Marimastat (0.8 mg/mL), or a combination of both inhibitors injected separately (each one at 0.8 mg/mL). Thirty minutes before the injections, the analgesic tramadol was administered by the subcutaneous route at a dose of 50 mg/kg to reduce pain associated with tissue damage. At 24 h post-injection of venom, PBS or the inhibitors, mice were sacrificed by cervical dislocation. Immediately afterwards, the right gastrocnemius muscles were dissected and submerged in Zinc Formalin Fixative (Sigma-Aldrich, St Louis, MO, USA) for 24 h at 4 °C. Gastrocnemius muscle was selected because it has been the model used in our previous studies of muscle regeneration after myonecrosis induced by *B. asper* venom [6,7,8]. The tissue was dehydrated with serial ethanol and xylol and embedded in paraffin, and tissue sections of 4 µm thickness were obtained from the mid-belly section of the muscle.

Tissue was oriented to obtain transverse sections, deparaffinized with xylene, hydrated in distilled water, and stained with hematoxylin and eosin for microscopic evaluation. The totality of each cross-section of muscle tissue was captured in 6 to 9 non-overlapping images using a Delphi-X Observer light microscope (Euromex, Arnhem, The Netherlands) with a camera. The total muscle area and the areas of necrotic muscle, i.e., areas with fibers showing hypercontraction of myofibrils, characterized by fragmentation of the cytoplasmic material, whereby masses of myofilaments and empty spaces are observed inside damaged fibers, were measured. Areas of necrosis and non-damaged muscle were identified by the analysts by visual inspection using Image Focus Alpha version x64, 1.3.7.16909.20200404 (Euromex). The extent of muscle damage was expressed as the percentage of the areas of myonecrosis relative to the total muscle area.

### 4.4. Effect of Varespladib and Marimastat on Muscle Regeneration After Myonecrosis Induced by B. asper Venom

For the evaluation of the effect of Varespladib and Marimastat on muscle regeneration, groups of 5–7 mice received an i.m. injection of either 50 µL PBS or 50 µg venom, dissolved in 50 µL PBS, as described above. Seven mice were used in the case of experiments where samples were collected at 28 days in order to have a minimum of five mice per group, because some mice died after 14 days. Thirty minutes before venom or PBS injection, tramadol was administered, as described above. Twenty-four hours after venom or PBS injection, mice received an i.v. administration of 100 µL of either PBS, Varespladib (0.8 mg/mL), Marimastat (0.8 mg/mL), or a combination of both inhibitors injected separately (each one at 0.8 mg/mL). Inhibitors were dissolved first in DMSO (20 mg/mL) and diluted in PBS to reach the final concentration used. Mice were sacrificed by cervical dislocation 14 and 28 days after venom injection, and the right gastrocnemius muscles were dissected for tissue processing, as described above. In samples collected 28 days after venom injection, the wet weights of the injected gastrocnemius muscles were determined before tissue processing. In order to assess whether the inhibitors had any effect on skeletal muscle 28 days after injection, control groups of mice received an i.m. injection of PBS followed 24 h later by an i.v. injection of either Varespladib, Marimastat, or Varespladib + Marimastat. Mice were sacrificed as described above after 28 days and tissues were processed for histological evaluation.

For the analysis of muscle regeneration, samples of tissue collected 14 and 28 days after envenoming were analyzed. These time intervals were selected on the basis of previous studies of muscle regeneration after necrosis induced by *B. asper* venom [6,8]. The extent of muscle regeneration was expressed in two ways: (a) the total number of regenerating muscle fibers in the total area of the muscle section and (b) the diameters of all regenerating muscle fibers. The totality of each cross-section of muscle tissue was captured in several images for the analyses. Regenerating muscle fibers were identified as those having centrally located nuclei, which is a morphological feature of regenerating fibers that is maintained for a prolonged period after the onset of necrosis induced by a snake venom toxin [42]. For these quantitative assessments, muscle fibers with centrally located nuclei were identified by visual inspection and counted, and their diameters were measured manually and counted using Delphi-X Observer (Euromex) and the Image Focus Alpha software (Euromex).

For the detection of fibrotic areas in muscle, 4 µm muscle sections of tissue processed as described above were stained for one hour with a 0.1% (*w*/*v*) Sirius red, C.I. 35780 (Polysciences Inc., Warrington, PA, USA), dissolved in saturated aqueous picric acid (Sigma-Aldrich), following the protocol of Dapson et al. [43]. The slides were dehydrated with three changes of 100% ethanol, cleared in three changes of 100% xylene, and mounted in Dako Mounting Medium (Statlab Medical Products, McKinney, TX, USA). Micrographs covering the totality of each muscle tissue section were acquired using the Delphi-X Observer microscope (Euromex). The total cross-sectional muscle area was measured, along with regions in which fibrosis predominated, defined as areas of damaged muscle within muscle fascicles staining positively with picrosirius red. Staining corresponding to dense fibrous connective tissue located between muscle fascicles was excluded from the analysis. Areas of intramuscular fibrosis were identified exclusively by manual visual inspection by the observers, and their surface area was subsequently measured using ImageJ software, version 1.54i (NIH, Bethesda, MD, USA). The extent of fibrosis was expressed as the percentage of the area within muscle fascicles with predominant staining with picrosirius red relative to the total area of the tissue examined. In the study of hematoxylin–eosin and picrosirius-stained sections, samples were anonymized, and the analyses were carried out simultaneously by two blinded observers. Discrepancies were discussed and consensus decisions were made.

### 4.5. Statistical Analysis

Graphics for the figures were made using the program RStudio version 4.5.1 (13 June 2025) (R foundation). Differences between experimental groups were assessed using a one-way ANOVA followed by Tukey post hoc test for normally distributed data. Non-normally distributed data were analyzed using nonparametric tests depending on the characteristics of the distribution. In the case of the percentage of fibrosis at 14 and 28 days, a generalized linear model (GLM) with Gamma distribution was used, because the data consisted of continuous positive values, including decimals, and showed a right-skewed distribution. For the analysis of the number of fibers with centrally located nuclei at 14 days, the data showed a right-skewed distribution with extreme overdispersion; therefore, a negative binomial regression model was used.

## Data Availability

The original contributions presented in this study are included in the article/Supplementary Material. Further inquiries can be directed to the corresponding authors.

## Supplementary Materials

The following supporting information can be downloaded at: https://www.mdpi.com/article/10.3390/toxins18090359/s1. Figure S1: Wet weight (g) of the right gastrocnemius muscle of CD1 mice 28 days after intramuscular injection of 50 µg *B. asper* venom, dissolved in 50 µL PBS, followed, 24 h later, by the intravenous administration of either PBS (Ba), Varespladib (Ba + Var), Marimastat (Ba + Mar) or Varespladib + Marimastat (Ba + Var + Mar). The bar of PBS corresponds to non-envenomed mice receiving PBS. Wet weight of envenomed mice receiving Marimastat was significantly higher than that of envenomed mice not receiving inhibitors (* *p* < 0.05). Statistical analysis was performed using one-way ANOVA with Tukey’s *post hoc* test. The weight of the muscle of mice injected with PBS only was significantly different from Ba (*p* < 0.001), Ba + Var (*p* < 0.05) and Ba + (Var + Mar) (*p* < 0.05). No significant difference was observed between the treatments Ba + Mar and PBS (*p* = 0.44). Table S1: Raw data of the experiments performed in this study.
